# Supplementary material for: Are carbon emissions trading and green financial instruments synergistic? -Comprehensive quantitative research based on content analysis
Source: PLoS One. 2024 Mar 7;19(3):e0298601. doi: 10.1371/journal.pone.0298601 (PMC10919601; doi:10.1371/journal.pone.0298601)
Supplement: S1 Data — (ZIP) [file pone.0298601.s001.zip › Data/地级市Z_F工作报告.zip!/║■▒▒/╧╠─■╩╨/└┤╫╘╣1⁄2╓┌║┼í╛┬φ┐╦╩2╛▌═°í┐_2009.docx]

各位代表：

　　现在，我代表市人民政府，向大会报告工作，请予审议，并请各位市政协委员和列席人员提出意见。

　　一、2008年工作回顾

　　2008年是极不平凡的一年。面对历史罕见的冰雪灾害、 严峻复杂的经济形势，在省委、省政府和市委的坚强领导下，全市上下深入贯彻落实科学发展观，团结拼搏，克难奋进，推动经济稳健运行，保持社会安定和谐，在鄂南经济强市建设的进程中迈出了坚实步伐。除居民消费价格指数外，本届人大一次会议确定的年度主要预期目标全面超额完成。

　　经济发展跃上了新台阶。全市生产总值达到359.19亿元，增长16.1%，增幅居全省第一位；地方财政总收入完成26.56亿元，增长28.6%，其中地方一般预算收入完成14.33亿元，增长29.1%，增幅居全省第二位。工业经济提速提质。规模以上工业企业新增91家，达到627家；规模以上工业增加值达到113.41亿元，增长30.5%，增幅居全省第三位；工业经济效益综合指数211.18，提高3.96个百分点。农业丰产丰收。实现农业总产值134.78亿元，增长17.7%。粮食总产达到20.55亿斤，增长14%。特色农业板块基地面积新增30万亩，年销售收入过亿元和省级农业产业化龙头企业分别达到14家、17家。低丘岗地改造完成31.6万亩，占全省的三分之一强。第三产业发展加快。社会消费品零售总额达到136.81亿元，增长29.2%，增幅居全省第一位。接待游客560万人次，实现旅游收入20.7亿元，分别增长30%和37.5%。金融机构各项存款余额278.93亿元，各项贷款122.03亿元，分别增长23.8%和5.9%。完成房地产投资25.35亿元，增长73.6%。保险、信息、中介等服务业稳步发展。县域经济实力增强。6个县（市、区）完成工业增加值95.6亿元，增长32.1%；地方一般预算收入首次全部突破亿元大关。民营经济不断壮大。二、三产业民营经济增加值达到220.76亿元，增长29.8%，占全市生产总值的比重达到61.5%，提高2.2个百分点。

　　城乡面貌发生了新变化。基础设施、生态环境等方面的投入力度加大。全社会固定资产投资达到200.83亿元，增长45.1%，增幅居全省第一位，其中城镇以上固定资产投资达到164.52亿元，增长49.8%，增幅居全省第二位。城镇建设扎实推进。咸宁、赤壁、嘉鱼荣获全省城镇规划建设管理“楚天杯”和省级文明城市（县城）称号。“百镇千村”示范工程和“清洁家园”建设稳步推进。新建通村公路1724公里，新增户用沼气池1.2万口，新解决17.3万人的饮水困难。节能环保工作取得明显成效。重点流域区域污染防治得到加强，依法关闭小造纸、小炼钢、小炼铁企业30家，万元生产总值综合能耗和化学需氧量、二氧化硫排放量年度节能减排目标任务可超额完成。

　　改革开放实现了新突破。“两型”社会建设综合配套改革试验开局良好，三年行动计划全面启动。农村公益性服务“以钱养事”机制进一步完善，农业“小三场”和集体林权制度改革稳步推进，粮食流通、农资购销等体制改革不断深化。政策性“三农”保险试点、农村集体资产产权制度创新试点有序推进。重点企业改制重组后活力增强。部门预算制度渐趋规范，税收征管机制日益完善。国有资产管理进一步加强。投资、融资、用地、行政管理等方面体制机制不断创新，科技、教育、文化、卫生等领域的改革逐步深化。积极参加鄂港粤、鄂沪贸洽会、中博会、厦门投洽会、深圳高交会等大型招商活动，光彩事业咸宁行活动成果丰硕，融入武汉城市圈步伐加快。招商引资项目达到395个，到位资金82.5亿元，增长42.7%。7个省级开发区和工业园引资到位41.6亿元，增长28.9%，其中咸宁经济开发区增长61.6%。对外开放不断扩大。实际利用外资达到9641万美元，增长35.4%。外贸出口总额达到1.09亿美元，增长36.8%。

　　社会事业取得了新进步。基础教育、职业教育、高等教育[FS:PAGE]稳步发展，城乡义务教育免收学杂费工作全面落实，困难学生资助力度不断加大。科技创新能力增强。争取省级以上科技项目31个，引进、推广科技成果100余项，高新技术产业增加值达到12.8亿元，增长28.8%。社会科学、文学艺术、新闻出版、广播电视等事业繁荣活跃，精神文化产品不断涌现。咸宁市歌舞团正式组建。中央电视台《同一首歌》走进咸宁，加强了对咸宁的宣传推介。以迎奥运为主题的群众性体育活动广泛开展。新型农村合作医疗保险全面推进，农民参合率达到85.4%。城镇居民基本医疗保险试点顺利开展。手足口病、艾滋病、血吸虫病等重大传染病和地方病防治取得实效。低生育水平持续稳定，人口自然增长率控制在5.7‰以内。对台、外事、民族、宗教等工作扎实推进。人防、保密、气象、水文、防震等工作得到加强。人事、编制、统计、物价、审计、档案、方志等工作取得新的成绩。妇女、儿童、老龄、残疾人等事业全面发展。

　　人民生活有了新改善。就业再就业工作不断加强。城镇就业新增39985人，下岗失业人员再就业新增11312人，城镇登记失业率控制在4.2%以内。社会保障体系不断完善。社会保险扩面新增5.55万人次。救灾济困力度加大。在全省率先完成5281户因灾倒损民房的恢复重建任务，改造农村特困户危房3747户，救助受灾群众18万人，新增农村低保2.1万人。发放城乡困难群众大病医疗救助资金2093万元、低保家庭价格补贴837万元。新建经济适用房13.05万平方米，发放廉租房租金补贴76.29万元。扶贫开发、库区移民等工作得到加强。城镇居民人均可支配收入、农民人均纯收入分别达到10597元和4411元，分别增长14.9%和18%。市政府年初承诺的十件实事全面落实。

　　民主法制和政府执行力建设得到了新加强。基层民主政治建设稳步推进，村委会换届选举顺利完成。依法行政工作全面推进，“五五”普法教育有序开展。人大代表建议和政协委员提案全面办结。科学民主决策机制不断完善，重大事项集体决策、专家咨询、社会公示和听证、合法性审查等制度得到落实，新聘请了10名省内知名专家、学者担任市咨询委员会委员。政务公开稳步推进。精神文明创建活动深入开展。应急救援和减灾防灾体系逐步健全，信访、维稳工作进一步加强，安全生产、社会治安形势总体趋好，“平安咸宁”创建成效明显。国防动员、民兵预备役和“双拥”工作得到加强。行风评议、文明执法教育和“一转变两提高”等活动深入开展，行政效能有了新的提升。源头治腐力度加大，政府系统党风廉政建设得到加强。

　　过去一年，我们经受了考验。面对五十年一遇的冰雪灾害，及时启动应急预案，组织动员各方面力量，千方百计保障人民群众正常的生产生活秩序。面对四川特大地震灾害，全市人民以大爱无私的精神，积极支持灾区救灾重建工作，出资出力，抢前争先，率先在全省完成了援建工程任务。面对国际金融危机的冲击，沉着应对，化“危”为“机”，保持了经济平稳较快增长。

　　过去一年，我们收获了欣喜。鄂南经济强市的建设目标，绘就了我市跨越发展的宏伟蓝图。学习讨论和外出考察活动的组织开展，核电和核技术产业高层论坛的成功举办，掀起了新一轮思想解放的热潮。改革开放三十周年和建市十周年庆祝活动的隆重举办，激发了全市干部群众投身改革发展的极大热情。全省雪灾倒损民房恢复重建、“双低”改造、秋冬农业开发和旅游工作等现场会在咸宁的召开，鼓舞了我们争先创优的斗志。特别是深入学习实践科学发展观活动的扎实开展，更加坚定了我们推进科学发展的信心和决心。

　　回顾过去一年的工作，我们深切体会到，做好新形势下的政府工作，必须坚持好中求快、科学发展，任何时候都不可动摇；必须坚持抢抓机遇、奋勇争先，任何时候都不可懈怠；必须坚持深化改革、扩大开[FS:PAGE]放，任何时候都不可停步；必须坚持以人为本、共建共享，任何时候都不可忘却；必须坚持统筹兼顾、整体推进，任何时候都不可偏颇。

　　回顾过去一年的工作，我们清醒认识到，我市经济总量不大与结构不优的问题同时存在，农业综合生产能力较弱与农民稳定增收机制尚未根本建立的问题同时存在，部分企业融资难与产业集中度不高的问题同时存在，社会事业发展相对滞后与社会保障能力较弱的问题同时存在，政府职能转变不到位与少数公务人员作风不实的问题同时存在，等等。我们将倍加珍惜当前的良好局面，倍加重视前进中的困难与问题，倍加努力改进政府工作，不断开创咸宁改革发展的新局面。

　　各位代表！过去的一年，挑战非同寻常，成绩来之不易。我们取得的每一项进步、每一点成绩，都归功于党中央、国务院和省委、省政府的亲切关怀，归功于市委的坚强领导，归功于市人大、市政协的监督支持，归功于全市人民的团结拼搏。在此，我谨代表市人民政府，向全市广大干部群众，向各位人大代表、政协委员和各民主党派、工商联、无党派人士，向离退休老同志，向驻咸解放军、武警官兵、政法干警，向所有关心、支持咸宁发展的社会各界人士和海内外朋友，表示最崇高的敬意和最衷心的感谢！

　　二、2009年的形势和任务

　　2009年是新中国成立六十周年，是实现“十一五”发展规划的攻坚之年，是建设鄂南经济强市的关键之年，是应对国际金融危机、推进科学发展的重要之年。做好今年的政府工作，意义十分重大。

　　今年，我们面临的经济形势异常复杂、异常严峻。国际金融危机尚未见底，对实体经济的影响逐步加深。主要发达国家经济仍可能全面衰退，新兴市场国家和发展中国家经济增速将明显放缓。国际市场需求减弱，势必加剧国内市场竞争，给咸宁的经济社会发展带来更大的挑战。受成本上升和外需收缩的双重影响，我市企业生产经营的压力进一步增大；受增值税转型和税负政策调整的双重影响，我市财政增收的压力进一步增大；受农民工部分返乡和市内待岗群体惯性增加的双重影响，我市就业工作面临的压力进一步增大；受不稳定因素增多和利益关系调整的双重影响，我市维稳工作的压力进一步增大，等等。我们将进一步增强忧患意识和责任意识，把困难估计得更充分一些，把措施考虑得更周密一些，把工作做得更扎实一些，防止由于估计不足和准备不够而陷入被动。

　　纵观全局，咸宁的发展仍处于重要战略机遇期。我国经济发展的基本面依然向好。改革开放三十年的厚实积淀，为抗御风险提供了有力支撑。金融危机形成的“倒逼机制”，为加快转变发展方式提供了难得机遇。我国工业化、城镇化快速推进，政局稳定，市场广阔，发展潜力很大。中央实施积极的财政政策和适度宽松的货币政策，出台一系列扩大内需的重大措施，沿海产业向中西部地区加快转移，促进中部地区崛起战略全面推进，武汉城市圈“两型”社会建设综合配套改革试验启动实施，为我们深化改革、扩大开放、加快发展、改善民生带来了新的更大机遇。

　　正视自身，咸宁的发展具有诸多基础优势。咸宁有着良好的地理区位、人文山水、交通能源等基础条件，有着明显的后发优势，既具有深化改革、扩大开放的综合优势，又具有发展内生型经济的比较优势；既具有“两型”社会建设综合配套改革试验的“特区”政策，又具有中央、省委在我市开展深入学习实践科学发展观活动试点工作的“小灶”待遇，形成了相对特殊的“小气候”，为争取国家和省里的支持提供了契机，为破解经济发展瓶颈制约、探索建立有利于科学发展的体制机制创造了条件。改革开放以来，特别是建市十年来，咸宁人民在历届党委、政府的领导下，励精图治，艰苦奋斗，取得了令人瞩目的成就，为实现科学发展、跨越发展奠定了坚实基础。

[FS:PAGE] 　　展望来势，咸宁的发展进入了全面提速、重点跨越的新时期。综合实力明显提升，基础设施明显改善，持续发展能力明显增强。在2008年底中央新增千亿元投资中，我市争取到176个项目，投资规模达到10.78亿元。在全省2009年底前拟新开工的重大项目清单中，我市上榜项目110个，投资规模达到696.6亿元，占全省7250.2亿元的9.6%。特别是一批重大项目，如咸宁核电站、蒲圻电厂二期、武咸城际铁路、杭瑞高速咸宁段、大广高速通山段和林浆纸一体化项目的开工建设，展示了咸宁良好的发展预期。我们完全有理由相信：咸宁的明天一定会更加美好！

　　面对挑战，信心比黄金更宝贵；面对机遇，行动比创意更紧要。有信心，就有凤凰涅槃、浴火重生的勇气；就有迎接挑战、战胜困难的力量；就能够把广大干部抓经济工作的积极性、主动性、创造性充分调动起来，把投资者的投资热情焕发出来，把人民群众对经济发展的良好心理预期提振起来。只要我们坚定信心和决心，不为任何风险所惧，不为任何干扰所惑，就能在应对挑战中把握机遇，在战胜困难中赢得主动，在开拓进取中增创优势。

　　2009年政府工作的总体思路是：全面贯彻党的十七大、十七届三中全会、省委九届六次全会和市委三届六次全会精神，以开展深入学习实践科学发展观活动和“两型”社会建设综合配套改革为契机，以全面推进科学发展、建设鄂南经济强市为主线，以保增长、扩内需、调结构、重民生、促和谐为主要任务，以四个“十大建设工程”（十大基础设施建设工程、十大产业项目建设工程、十大生态环境建设工程、十大民生建设工程）为重要抓手，着力推进产业优化升级和发展方式转变，着力推进改革创新和开放合作，着力推进城乡协调发展和民生事业建设，努力推动经济社会又好又快发展。

　　全市经济社会发展的主要预期目标是：生产总值增长15%以上，万元生产总值综合能耗下降4.9%，化学需氧量和二氧化硫排放量分别下降2%、4%；全社会固定资产投资增长30%以上，社会消费品零售总额增长23%，吸收外商直接投资增长15%，外贸出口增长15%；地方一般预算收入增长18%；城镇居民人均可支配收入增长8%，农民人均纯收入增长9%；居民消费价格涨幅控制在4%左右，城镇登记失业率控制在4.5%以内，人口自然增长率控制在6‰以内。确定以上目标，是实现保增长、促发展的需要，是衔接和完成“十一五”发展规划的需要，是发挥后发优势、加快建设鄂南经济强市的需要。在实际工作中，我们将本着“跳起来摘桃子”的精神，积极主动工作，力争取得更好的发展成果。

　　三、2009年的工作重点

　　围绕2009年的目标任务，我们将着力抓好以下重点工作：

　　（一）着力扩大投资和消费需求，切实保持经济平稳较快增长。全面落实国家扩大内需的政策措施，不断扩大投资需求和消费需求，以投资带动消费，以消费促进增长。

　　千方百计扩大投资规模。大力实施“项目建设年”活动，按照策划一批、开工一批、储备一批的要求，实现项目建设的有序接替、滚动发展，谋划和形成一批千亿元、百亿元、十亿元左右的重点建设项目梯队或产业集群。完成重点项目投资150亿元，带动全社会固定资产投资300亿元以上。全力推进武咸城际铁路、武咸快速通道、杭瑞高速咸宁段、大广高速通山段、咸楠高速、赤壁快速通道、武赤线陶嘉一级公路、市区环城公路、武咸天然气管道、向家坝至上海高压直流输电咸宁段等“十大基础设施建设工程”，同时协助做好鄂州至通山、咸宁至宜春铁路咸宁段，黄石至咸宁、咸宁至嘉鱼及仙桃高速公路的前期工作。完善重大项目建设推进机制，确保早建设、早竣工、早投产、早见效。

　　千方百计扩大消费需求。努力增加城乡居民特别是中低收入者的收入，提高消费能力，稳定消费预期。完善促进消费的政策[FS:PAGE]措施，拓宽消费领域，改善消费结构，培育消费热点，推动消费持续升温。稳步促进教育、旅游、文化、网络和体育健身等热点消费，大力发展社区商业、物业、家政等服务性消费，适当扩大公益事业消费。开拓农村市场，扩大农村消费需求。大力实施“万村千乡市场工程”和“新农村现代流通服务网络工程”，鼓励连锁超市下乡、家电下乡。支持兴办集村务、商务、服务于一体的农村综合服务社。稳健发展房地产市场，加快廉租房等保障性住房建设，支持中小户型、中低价位普通商品房开发。加强市场监管，搞好消费维权，让群众放心消费。

　　千方百计保障资源要素供给。加强经济运行调度，帮助企业做好煤、电、油、气、水、运等各类生产要素的供应。建立领导联系企业、部门服务企业制度，跟踪分析重点产业、重点企业、重点产品的运行态势，帮助解决企业生产经营的困难和问题。加强企业周边社会治安管理，抓好软硬环境建设。抢抓国家产业振兴发展机遇，研究出台具体措施，支持企业加快发展。

　　（二）着力推进结构调整，加快转变发展方式。把保增长、扩内需与调结构有机结合起来，加快推进发展方式转变，增强发展的协调性和可持续性。

　　大力推进产业结构调整优化。推动产业优化升级，培育壮大产业集群。每个县（市、区）突出抓好1—2个产业集群。通过重大项目的实施，带动优势产业的壮大，确保全市规模以上工业增加值增长20%以上。在抓好已有产业项目的基础上，重点推进“十大产业项目建设工程”，即推进以咸宁核电开工及蒲圻电厂二期为主的电力能源项目建设；以林浆纸一体化及板材集群发展为主的森工造纸项目建设；以红牛饮料二期、维雪啤酒搬迁扩产、黄鹤楼酒业及农副产品加工为主的食品饮料项目建设；以蒲纺省级经济开发区创建争取、天化麻业二期、银泉维新技改、洁丽雅纺织扩产、元田制衣为主的纺织服装项目建设；以华新水泥二期、通城100万吨粉磨站和咸安、嘉鱼、崇阳、通山新型干法水泥及钒矿、锰矿、高岭土等资源开发为主的建材冶金项目建设；以三环方向机搬迁扩产、合加环保设备、IT电子、涂附磨具和西门子配套产业为主的机械电子项目建设；以簰州湾天然气勘探、精细化工园区规划和天然气利用为主的燃气化工项目建设；以温泉旅游生态新城、八一温泉度假村、咸安金桂湖二期、赤壁龙佑二期、赤壁古战场二期、嘉鱼山湖温泉度假村、通山富水乐园等为主的生态旅游项目建设；以市县城区房地产开发为主的房地产项目建设；以广东工业园和武汉园外园为主的园区项目建设。促进开发区、工业园区体制机制创新，完善园区基础设施，优化园区功能布局，引导产业和企业向园区聚集。大力提升以旅游业为主体的服务业。坚持以生态为基础、以文化为核心、以产业为引擎，推动旅游与工业、农业、文体和商务等产业有机结合。加快旅游基础设施建设，加大旅游行业创优、旅游名镇名村创建力度，提升旅游服务质量。加强旅游产品宣传促销，筹办首届中国咸宁温泉国际旅游节，确保接待游客增长21%，旅游收入增长35%。运用现代服务技术和经营方式，改造提升商贸、餐饮等行业。充分发挥区位和交通优势，大力发展现代物流业，建设区域物流配送中心。积极发展电子商务、科技服务、信息咨询等生产性服务业，发展创业投资、风险投资、信用担保等新兴行业，促进邮政通信业，发展鉴证评估等中介服务业，发展服务外包。进一步改善金融生态环境，推动金融、保险业健康发展。

　　大力推进企业结构和产品结构调整优化。加快企业结构调整，推进企业并购、重组、联合，扩大产能，延伸产业链，壮大产业群；进一步支持三环方向机、巨宁森工、嘉裕管业、汇美达、能一郎、精华纺织、华宁橡胶等企业做好上市工作；加快推进“中小企业成长工程”，培育一批咸宁工业代表队，[FS:PAGE]确保规模以上工业企业新增80家以上，2家以上企业进入全省“三个三工程”。加快产品结构调整，实施精品名牌战略，争取1—2个产品跻身中国驰名商标，培育一批地方名牌，发展一批具有竞争力的拳头产品。

　　大力推进区域协调发展。以市区为中心，着力打造由沿江、沿铁路、沿京珠高速连接的“北三县”中小城市群，沿杭瑞高速、沿国道连接的“南三县”特色经济带，构建以城带乡、南北互动、有机融合的区域经济发展格局。高昂市区发展龙头，按照“市区一体、互为依托、和谐发展、共同繁荣”的要求，整合市区资源，加快市区经济发展，力争主要经济指标增幅高于全市平均水平。加快工业新城建设，力争咸宁经济开发区当年签约、建设、投产项目分别达到20个，引进资金40亿元，实现税收3亿元。加大中心城区建设力度。完成城市总体规划修编，城区控制性规划覆盖率达到85%。完成市中心客运站、南外环、青龙路、武广客运站配套设施等一批续建项目，启动桂乡大道生态走廊建设，完成桂花中路、茶花路、南大街、城区自来水管网等改造项目，抓好城区停车场和3个城市公园建设，完善城市功能。深入开展全国文明城市、国家园林城市、国家卫生城市、中国优秀旅游城市“四城同创”活动，继续推进“洁、绿、亮、美”工程，推进城市管理综合执法，提升城市形象。加大城市经营力度，抓好土地、资产等资源的营运，走以城养城、以城建城、以城兴城的路子。协调推进县域经济发展。进一步简政放权，支持各县（市、区）发挥比较优势，走区域特色发展之路。鼓励争先进位，力争赤壁市进入全省县域经济前十名，咸安区、嘉鱼县进入全省第一方阵，通城县、崇阳县、通山县在全省位次明显前移。加快小城镇建设。坚持特色立镇、产业兴镇，着力培育一批经济强镇。突破制度障碍，促进农民稳定有序向城镇转移。

　　大力推进区域创新体系建设。突出企业技术创新主体地位，鼓励企业以市场为导向，围绕新产品、新技术、新工艺开发和科技成果转化应用，有针对性地开展产学研合作，促进企业信息化建设。鼓励和支持高新技术产业发展，力争省级以上高新技术企业数增长50%，高新技术产业增加值增长30%以上。完善技术创新激励机制，引导创新要素向企业、产业集中。创新科技投入方式，发挥政府投入的引导作用，加强高新技术产业示范基地、成果转化基地和科技企业孵化器建设。落实支持自主创新的政策措施，加强知识产权保护力度。加快经营管理人才、专业技术人才、高技能人才以及技能紧缺人才的培养、引进和使用，为推动自主创新提供有力的人才支撑。加强公民科学素质建设，培育全社会的创新精神。

　　大力推进节能减排和生态环境建设。按照“硬标准、硬约束、硬检验”的要求，强化节能减排工作责任制，严格执行项目开工建设环评审批和节能评估审查制度，严格控制高耗能、高排放行业的发展。大力发展循环经济，建设一批资源节约、环境友好型的示范园区、企业和村镇。加强冶金、建材、化工、电力等重点行业的节能改造，继续开展“九小”专项治理，大力推进全民节能行动。推进生态咸宁建设，重点实施咸宁垃圾焚烧发电、淦河生态修复与综合治理、潜山国家森林公园二期、城市污水处理、崇阳凯迪生物质发电、赤壁城市防洪、陆水节堤航电枢纽、陆水流域生态环境保护、梓山湖生态科技新城、农村“清洁家园”建设等“十大生态环境建设工程”。积极开展西凉湖和斧头湖重点水域防治，加强天然林保护、退耕还林、自然山体水体保护、水土保持、用水水源地保护等工作。

　　（三）着力做好“三农”工作，扎实推进新农村建设。贯彻落实党的十七届三中全会精神，按照省委提出的“农村面貌三年实现明显变化，五至七年实现大变化”的要求，总结推广官桥八组新农村建设经验，全面推进农村改革[FS:PAGE]发展，促进农业持续增效、农民稳定增收、农村面貌不断改善。

　　进一步发展现代农业。稳定粮食生产，建设10万亩高产农田，力争粮食总产增长2%以上，确保主要农产品生产稳定。坚持开发与保护相结合，推进“在山上再造一个咸宁”战略，改造低丘岗地30万亩、低产林10万亩。大力发展规模农业、特色农业、品牌农业、效益农业，突出抓好蔬菜、果茶、马铃薯、畜禽、水产、速生丰产林等特色板块建设，力争全市特色板块面积新增20万亩，产值增长8%。加快推进重庆美多、中粮储、精武鸭、康誉食品、鹏程林农等一批农产品加工项目，重点培植一批带动力强的龙头企业，新增一批省级龙头企业，实现国家级龙头企业零的突破。创新农业产业化经营模式，推动金融资本、产业资本和技术要素以股权形式进入农业。在税收、信贷、用地、用电等方面，支持龙头企业加快发展。大力推进农科教结合，抓好农业科技成果转化和实用技术推广，提高农业机械化水平，增强农业综合生产能力。

　　进一步稳定和完善农村基本经营制度。赋予农民更加充分而有保障的土地承包经营权，稳定现有农村土地承包关系并保持长久不变。按照依法、自愿、有偿原则，引导土地承包经营权向种养大户、农民专业合作组织、龙头企业有序流转，发展适度规模经营。引导农民专业合作组织加快发展，提高农民组织化程度。

　　进一步促进农民增收。认真落实国家较大幅度增加农业补贴、扩大补贴范围、较大幅度提高粮食最低收购价、解决农民工就业等一系列强农惠农政策，拓宽农民增收渠道。大力发展观光农业、庭院经济，加快发展农村二三产业，完善企业、基地与农户的利益链接机制，扩大订单生产，带动农民增收。坚持农民外出务工与市内就业相结合，做好市内企业用工与返乡农民工就业的对接服务工作。加强市场监控，稳定农业生产资料和农产品价格。搞好农产品购销和调运，畅通流通渠道。加强农民负担监管，整治涉农乱收费行为。继续推进新农村示范村建设，实施扶贫整村推进，抓好通山县扶贫开发与新农村建设相结合示范工程。注重解决好陆水水库、咸宁核电移民安置户的生产生活困难。

　　进一步增加“三农”投入。完善支农资金稳定增长机制，进一步加大财政对“三农”的扶持力度。整合涉农资金，提高资金使用效益。探索以奖代补、先建后补、以物抵资、民办公助等方式，引导社会资金投入和农民投工投劳，推进农村各项建设。进一步发展农村社会事业，健全农村科技、教育、文化、卫生等服务体系，完善农村社会保障体系，扩大农业保险覆盖面。抓好病险水库除险加固、大型排涝泵站更新改造、大中型灌区续建配套及末级渠系建设。继续完善配套水、电、路、沼气等基础设施，实施“百镇千村”示范工程，不断改善农民生产生活条件和农村面貌。

　　（四）着力深化改革开放，不断增强发展活力。抢抓武汉城市圈“两型”社会建设综合配套改革试验机遇，坚定不移地实施开放先导战略，解放思想，先行先试，深化改革，扩大开放，增强发展的动力和活力。

　　加大“两型”社会建设综合配套改革力度。抓紧实施“两型”社会建设综合配套改革试验实施方案和六个专项规划，稳步推进三年行动计划，将重点改革任务分解落实到市直有关部门和各县（市、区），推动改革试验具体化、模块化、项目化，切实形成点面结合、上下联动、整体推进的工作格局。按照“五个一体化”和先行先试的要求，在观念、体制、产业、市场、基础设施、城乡统筹、环境保护和生态建设等方面，加快推进与武汉城市圈的全面对接。加快引进新型股份制商业银行、外资金融机构、证券机构来咸设立分支机构。全方位推进市厅、市校等方面合作，争取更多更好的政策、项目支持。

　　加大重点领域和关键环节改革力度。积极引导企业建立现代企业制[FS:PAGE]度，巩固和发展企业改制成果。完善国有资产监管体系，加快政企、政资、政事、政府与社会中介组织职能分开步伐。稳步推进事业单位分类改革。深入推进农村综合配套改革，巩固和完善农村公益性服务“以钱养事”机制。加快建立商业性金融、合作性金融、政策性金融相结合的农村金融体系，大力发展村镇银行，开展小额贷款公司试点，允许有条件的农民专业合作社开展信用合作。深入推进集体林权制度改革，基本完成集体林权确权到户任务。继续推进城中村、城郊村、园中村集体资产产权制度创新。进一步完善国有农场体制改革。推进财税、金融、投资、土地、价格等方面的体制机制创新。坚持依法治税理财，深化财税改革，完善征管机制，规范部门预算，优化支出结构。严格执行收支两条线规定，做好增值税转型和燃油税改革的实施工作。发挥财政资金的政策引导作用，通过资本注入、风险补偿等方式，加大对信用担保公司的支持力度。搭建银企对接平台，完善合作机制，着力解决中小企业贷款难题。围绕重大项目建设，创新投资融资机制。建立健全矿产资源有偿使用制度和生态环境补偿机制。优化土地利用结构和布局，完善土地投资强度分级分类管理制度，坚持节约集约用地，坚守耕地红线，提高土地利用效率。加强价格监测预警，发挥价格杠杆促进经济发展和结构调整的作用。

　　加大招商引资和对外开放力度。完善政策措施，优化发展环境，广招天下客商，承接产业转移。在符合国家产业政策和生态环保要求的前提下，不论轻工业与重工业，不论技术含量高中低，不论企业规模大与小，都大力引进，鼓励发展。创新招商方式，鼓励联合招商，巩固小分队招商，推进产业链招商、产业群招商。精心组织重大招商活动，着力招引现代农业、现代制造业、高新技术产业、现代服务业、基础设施建设领域的大项目，着力招引有利于促进产业聚集的龙头项目，着力招引高就业、高税收项目和循环经济项目。高度重视外资引进工作，提高外资利用质量。积极实施市场多元化战略，巩固传统市场，开拓新兴市场，推动出口稳步增长。加大对重点产业、企业和产品的支持力度，抓好一批出口基地建设。优化出口结构，提高出口产品质量、规模和档次。重点支持纺织、机电、农副产品深加工等优势产品出口。大力实施“走出去”战略，支持优势企业到境外投资办厂，扩大境外资源合作开发、工程承包、海员外派和劳务合作。

　　加大促进全民创业工作力度。研究出台《关于促进全民创业的实施意见》，引导高校毕业生自主创业、灵活就业，推动城乡居民创业，支持各类企业再创业，让全民创业、自主创业成为全市人民的共同追求。进一步放宽市场准入，完善体制机制，支持和引导个体私营等非公有制经济进入公用事业、基础设施、金融服务、高新技术产业、特色优势产业、劳动密集型产业、生态建设以及社会事业等领域，促进非公有制经济健康快速发展。大力实施“回归工程”，鼓励、支持外出务工人员回归创业，促进“打工潮”向“创业潮”转变。

　　（五）着力发展以改善民生为重点的社会事业，努力提高人民群众生活水平。围绕人民群众最关心、最直接、最现实的利益问题，着力保障和改善民生，加强社会建设和管理，促进社会和谐稳定。

　　高度重视就业工作。健全就业工作目标责任制，实施更加积极的就业政策，以创业带动就业，以培训促进就业，以开发公益性岗位扩大就业。切实抓好返乡农民工、高校毕业生、转业退伍军人、城镇困难居民等群体的就业工作，推动城乡劳动就业一体化。注重发挥人力资源市场在就业方面的基础性作用，注重加强劳务协作，广辟就业渠道。采取积极措施减轻企业负担，稳定企业用工，保持就业局势的基本稳定。

　　高度重视社会保障体系的完善。继续做好社会保险扩面征缴工作。启动[FS:PAGE]农业“小三场”、国有林场职工和村级主职干部参加基本养老保险工作，加快被征地农民基本生活保障制度建设，开展新型农村社会养老保险试点。完善覆盖城乡的基本医疗保障体系，扩大受益面，提高受益水平。确保城镇居民医疗保险参保率达到75%以上，新型农村合作医疗参合率达到90%以上。做好关闭破产企业退休人员参加医疗保险工作。完善城乡低保制度，做好社会救助工作，保障和改善困难群众基本生活。继续推进农村特困户危房改造。抓好社会福利中心、儿童福利中心、流浪未成年人救助保护中心建设。做好农村特困群众大病医疗救助和临时救助工作。启动0—6岁贫困残疾儿童抢救性免费康复工作。

　　高度重视社会事业的发展。实施教育优先发展战略，完善和巩固九年制义务教育，加大对高中阶段教育的支持力度，大力发展职业教育，努力提升高等教育、学前教育、特殊教育。抓好农村“普九”债务化解工作。落实义务教育学校绩效工资政策，使义务教育学校教师平均工资水平不低于当地公务员水平。积极做好咸宁职教园区建设项目的争取工作。深化文化体制改革，大力发展公益性文化事业和经营性文化产业，大力推进广播电视“村村通”、文化信息资源共享工程农村服务点、农村电影数字化放映等惠民工程建设。进一步发展社会科学、新闻出版、文学艺术等事业，加强文物和非物质文化遗产保护，挖掘地方特色文化资源。加强文史研究和档案、方志等工作。发展体育事业和体育产业，推进群众性体育活动蓬勃开展。深化医疗卫生体制改革，发展社区卫生服务，加强农村三级卫生服务网络建设，促进基本公共卫生服务均等化。继续抓好重大传染病的预防和监控，提高人民群众健康素质。加快实施妇女儿童发展纲要。重视和支持老龄、残疾人事业。加快推进市中心医院、博物馆、妇儿中心、青少年宫等基础设施建设。加强人口和计划生育工作，稳定低生育水平，控制出生人口性别比，提高人口出生素质。

　　高度重视农产品、食品药品质量安全和生产安全。推进农产品标准化生产，加强农产品安全监管。开展食品药品专项整治，严防重大食品药品安全事故的发生。认真落实安全生产责任制，加强管理监督和隐患排查，继续推进矿山、危化品、道路交通、地质灾害等重点领域的专项整治，坚决遏制重特大事故的发生。加强社会化消防管理，做好农村和社区防火工作。

　　高度重视社会稳定工作。建立以人民调解为基础的多元纠纷解决机制，健全信访工作责任制，开展大接访、大排查、大调处，妥善处理人民内部矛盾。完善应急反应机制，加强预警预测预防，做好防灾抗灾减灾救灾工作，积极预防和妥善处置突发事件。充分发挥气象、水文、防震等工作在防灾抗灾减灾救灾中的作用。加强国家安全和保密工作。加强社会治安综合治理，健全社会治安防控体系，深化“平安咸宁”创建活动，深入开展严打整治，依法打击违法犯罪，增强人民群众的安全感。

　　高度重视社会管理工作。创新社会管理体制机制，发挥各类社会组织的作用。完善城市社区功能，开展农村社区建设试点。发挥工会、共青团、妇联等人民团体在社会管理服务中的作用。加强基层民主政治建设，完善村(居)民自治，健全政务公开、厂务公开、村(居)务公开等制度。维护司法公正，支持审判机关和检察机关依法独立行使审判权和检察权。深入开展“五五”普法教育，认真做好法律服务和法律援助工作，大力推进“法治咸宁”建设。加强社会主义核心价值体系和思想道德建设，深入开展精神文明创建活动。深入开展国防教育，提高全民国防意识，支持驻咸解放军和武警部队建设，抓好民兵预备役和人民防空工作，积极开展双拥共建活动。依法加强对民族宗教事务的管理。注重发挥对台、外事等工作在经济社会发展中的职能作用，发挥人事、编制等工作在人[FS:PAGE]才人事工作中的基础作用，发挥统计调查工作的信息、咨询和监督作用。

　　（六）着力提高政府执行力、公信力，加快建设服务型政府。坚持把科学发展、跨越发展作为第一要务，把当好公仆、为民服务作为第一职责，把狠抓落实、苦干实干作为第一选择，把依法行政、廉洁从政作为第一准则，加强政府自身建设，提高人民群众对政府的满意度和信任度。

　　更加注重依法行政。坚持市委统一领导，自觉接受市人大的法律监督、工作监督和市政协的民主监督，认真听取各民主党派、工商联和无党派人士的意见，自觉接受新闻舆论和社会公众的监督。推进决策科学化、民主化，完善重大行政决策程序和相关制度，依法保障人民的知情权、参与权、表达权、监督权。建立健全规范性文件监督管理制度。严格依法行政，做到有权必有责、用权受监督、违法受追究、侵权须赔偿。改革行政执法体制，完善行政执法程序，规范行政执法行为，开展相对集中行政处罚权工作。

　　更加注重提高政府执行力。深化政府执行力建设，着力构建长效机制，不断提高政府效能。完善目标管理、绩效评估制度和竞争激励机制，严格落实行政首长负责制和行政问责制，全面推行岗位责任制、服务承诺制和限时办结制。坚持高标准、严要求，保持高效率、快节奏，做到工作程序简化、召开会议简短、文字材料简练、深入基层简从，把更多的时间和精力用于解决问题、推动发展。加大调查研究和监督检查力度，推动工作落实，保证政令畅通。注重加强对公务员的教育、管理和监督，提升能力，转变作风。

　　更加注重提高服务质量。加强行政服务网络建设，推进电子政务，加强信息公开。实行一站式、全程式、跟踪式、“保姆式”服务，拓宽投资项目绿色通道。推行企业设立和项目报建并联审批模式，做到一个部门牵头、一个窗口受理、一套数据审批、一次现场勘查、一次集中会审。建立服务企业“直通车”制度，切实为企业排忧解难。加强行政服务中心和综合招投标中心建设，完善部门协调配合机制，提高办事效率和服务质量。

　　更加注重廉政建设。推进政府系统惩治和预防腐败体系建设，加大对重点领域、重点部门、重点资金、重点项目的审计监督和行政监察力度，严肃查处腐败案件和失职、渎职行为。坚持厉行节约，严格控制一般性支出。加强政风行风建设，坚决防止和纠正损害群众利益的不正之风。

　　各位代表，努力为人民群众办好事、办实事，是各级政府应尽的职责。在这里，我代表市人民政府郑重承诺，今年我们将着力抓好“十大民生建设工程”：（1）筹措资金2200万元，新增农村劳动力转移培训3.6万人，其中免费开展为期3个月的培训1万人；新增城镇就业3万人，开发（保持）公益性岗位2000个。（2）筹措资金1.2亿元，建设廉租房14.4万平方米。（3）筹措资金800万元，新增农村低保1万人，提高农村五保户的供养标准。（4）筹措资金1.1亿元，建设农村公路500公里。（5）筹措资金380万元，建设农家书屋173个、乡镇综合文化站12个。（6）筹措资金1.02亿元，解决21.94万人农村安全饮水问题；对农民按规定购买家电实行13%的补贴。（7）筹措资金2000万元，支持1个县级医疗机构、10个乡镇卫生院和100个村卫生室建设，启动咸宁市中医院改造升级工程。（8）筹措资金3000万元，在咸宁新城区启动建设一所“九年一贯制”学校。（9）筹措资金500万元，为困难群众和农民工提供法律援助。（10）深化“双扶”活动，组织1000个机关单位结对帮扶村组和社区、30000名党员干部结对帮扶困难家庭；减少农村绝对贫困人口0.52万人、低收入贫困人口2万人。

　　各位代表！人民的信任和期待，是我们奋勇前行的强大动力。尽管前进的道路并不平坦，但我们有信心、有条件战胜一切艰难险阻。让我们紧密团结在以胡锦涛同志为总[FS:PAGE]书记的党中央周围，高举中国特色社会主义伟大旗帜，在市委的坚强领导下，进一步解放思想，抢抓机遇，同心同德，扎实工作，奋力谱写咸宁科学发展的新篇章，以优异的成绩向建国六十周年献礼!
